# Unilateral Oophorectomy and Age at Natural Menopause: A Longitudinal Community‐Based Cohort Study

**DOI:** 10.1111/1471-0528.17980

**Published:** 2024-10-10

**Authors:** Erin A. Brennand, Natalie V. Scime, Rebecca Manion, Beili Huang

**Affiliations:** ^1^ Department of Obstetrics and Gynecology University of Calgary Calgary Alberta Canada; ^2^ Department of Community Health Sciences University of Calgary Calgary Alberta Canada; ^3^ Department of Health and Society University of Toronto Scarborough Toronto Ontario Canada

**Keywords:** Alberta's Tomorrow Project, menopause, oophorectomy

## Abstract

**Objective:**

To determine the association between unilateral oophorectomy (UO) and age at natural menopause.

**Design:**

Secondary analysis of survey data from Alberta's Tomorrow Project (2000–2022).

**Setting:**

Prospective cohort study in Alberta, Canada.

**Population:**

23 630 women; 548 experienced UO and 23 082 did not experience UO.

**Methods:**

Flexible parametric survival analysis was used to analyse age at natural menopause, and logistic regression was used to analyse early menopause and premature ovarian insufficiency by UO status, controlling for birth year, parity, age at menarche, past infertility, hormonal contraceptive use and smoking.

**Main Outcome Measures:**

Age at natural menopause occurred by a final menstrual period without medical cause and sub‐classified as early menopause (< 45 years) and premature ovarian insufficiency (< 40 years).

**Results:**

Compared to no UO, any UO was associated with elevated risk of earlier age at natural menopause, which was strongest in early midlife (adjusted HR at age 40 1.71, 95% CI 1.31–2.19) and diminished over time. Compared to age 55 years at UO, risks of earlier age at natural menopause were largest and uniform in magnitude when UO occurred between approximately ages 20–40 years (adjusted HR for UO at age 30 2.32, 1.46–3.54) and then diminished as age at UO approached the average age at natural menopause. Any UO was associated with higher odds of early menopause (adjusted OR 1.90, 1.30–2.79) and premature ovarian insufficiency (adjusted OR 3.75, 1.72–8.16).

**Conclusions:**

Unilateral oophorectomy is associated with earlier age at natural menopause, particularly when performed before 40 years of age.

## 1 Introduction

Menopause is a consequence of ovarian ageing resulting in loss of ovulation and menstruation [1] Early menopause is defined as a final menstrual period prior to the age of 45 and is associated with numerous chronic health conditions including adverse cardiometabolic diseases [2, 3, 4], frailty and osteoporosis [4, 5], increased all‐cause mortality [6] and cognitive decline [7]. These risks are not necessarily attenuated by prescribed hormone therapy [8]. The timing of natural menopause varies across populations and is influenced by sociodemographic characteristics, as well as reproductive and lifestyle factors such as parity, smoking and age at menarche [9, 10, 11]. Bilateral oophorectomy induces immediate iatrogenic menopause and women undergoing this surgical menopause experience more extreme vasomotor symptoms, and have higher healthcare utilisation than women who undergo natural menopause [12]. Yet the impact of unilateral oophorectomy (UO) on the menopause transition is less clear, [13] and it is not known if some of the counselling for women undergoing risk‐reducing BSO [12] ought to be extended to this population as well.

It is historically assumed that UO negligibly affects ovarian function given that menstruation continues unaltered [14]. Yet the primordial follicle number is reduced by half following UO [13], which may theoretically accelerate the onset of menopause [15]. Population‐based studies appear to support this notion [16, 17, 18]. However, these studies have important limitations, namely the use of linear or logistic regression models for data analysis, which assumes a linear relationship between menopause timing and age at UO. As such, knowledge gap exists on how the timing of a UO impacts menopause over the full period of midlife ageing, and whether there is an age threshold where UO is less impactful on menopause timing. Moreover, suboptimal control for important confounding factors hampers the internal validity of studies to date.

This study explored the association of UO on age at menopause with a focus on exploring non‐linear and age‐dependent nuances in this association. Our hypothesis was that there may be a plateau in age at which point UO no longer is associated with menopause timing.

## 2 Methods

### 2.1 Study Design and Setting

We conducted a secondary analysis of the Alberta's Tomorrow Project (ATP), a province‐wide prospective cohort study aimed at investigating aetiology and healthcare utilisation related to cancer and chronic diseases [19]. Between 2000 and 2015, 34 950 English‐speaking women aged 32–71 years with no personal history of cancer were recruited into ATP using two‐stage telephone random digit dialling (from 2000 to 2008) and volunteer sampling (from 2009 to 2015). Self‐report questionnaires collected comprehensive demographic, health and lifestyle data at baseline and on an ongoing basis through follow‐up every 3–5 years. For this analysis, we used data from baseline questionnaires and all follow‐up questionnaires completed by August 2022 ranging from 1 to 5 study contacts.

### 2.2 Ethics Statement

The ATP study was approved by the Health Research Ethics Board of Alberta at Alberta Innovates (HREBA.CC‐17‐0461 and HREBA.CC‐17‐0494). This secondary analysis was approved by the Conjoint Health Research Ethics Board at the University of Calgary (REB22‐0742).

### 2.3 Inclusion and Exclusion Criteria

We included female participants who provided data on UO surgery, excluding participants with missing menopause data, unknown temporal order of UO and menopause, an extreme age at menopause (≤ 35 years or > 65 years) or missing covariate data (Figure S1).

### 2.4 Variables

The exposure was UO before menopause and age at the time of this procedure. Clinical indication for UO was not collected. The primary outcome was age at menopause and analysed as time‐to‐event outcome. Secondary outcomes were early menopause (final menstrual period < 45 years old) and premature ovarian insufficiency (POI; < 40 years old). Covariates were selected based on prior evidence and included: participant birth year, cigarette smoking status, age of menarche, parity, history of infertility and duration of hormonal contraceptive use in years [17].

### 2.5 Data Analysis

First, we analysed the association between UO and timing of natural menopause using flexible parametric survival analysis with non‐proportional hazards. That is, we allowed this association to vary over time using restricted cubic splines (one knot) to model the effect of UO across the range of possible ages of menopause. We counted person‐time at risk in years from age 35 to age at menopause or a censoring event, which were hysterectomy, a subsequent UO, attrition, last study contact or age 65 (whichever came first). We estimated crude cumulative incidence functions with simulation‐based 95% confidence intervals (CIs). We then estimated hazard ratios (HRs) and 95% CIs for earlier menopause adjusted for birth year, smoking (yes, never, previous smoker), age at menarche, parity, history of infertility (yes/no) and duration of hormonal contraceptive use (years). An HR > 1.0 indicates earlier time of menopause in those with UO compared to those without UO (the reference exposure group).

Next, we analysed the association between age at UO and timing to natural menopause among participants with UO before menopause using flexible parametric survival analysis with proportional hazards (low cell counts precluded modelling of a time‐varying effect). That is, we used restricted cubic splines to allow age at UO to have a non‐linear effect on the timing of menopause across the range of possible ages when UO occurred. Models were fit with 0–3 knots; Akaike information criterion (AIC) and precision of estimated parameters suggested that one knot at the 50th percentile was the best fit [20]. We estimated crude cumulative incidence functions and then estimated HRs and 95% CIs for earlier menopause with age 55 years at UO as the reference exposure group and adjusted for birth year, smoking, age at menarche, parity, infertility and duration of hormonal contraceptive use. We also predicted the median age at natural menopause from the adjusted model, where covariates were held constant at the median values for continuous covariates and the most prevalent category of categorical covariates [21].

Next, we analysed the association of UO with POI and early menopause using logistic regression among participants contributing person‐time up to 40 and 45 years, respectively. We estimated odds ratios (ORs) and 95% confidence intervals (CIs), unadjusted and adjusted for birth year, smoking, age at menarche, parity, infertility and duration of contraceptive use.

Finally, we performed three sensitivity analyses. The first restricted all models to those aged ≤ 60 years old at baseline to minimise potential exposure and outcome misclassification due to recall bias or memory error. The second restricted all models to participants who were administered one of the two baseline questionnaires that collected data on race/ethnicity and body mass index (BMI) to explore the potential confounding effect of race/ethnicity and BMI. Race/ethnicity was measured with binary indicators for Asian, Black, Hispanic, Indigenous, Middle Eastern and White based on self‐identified ancestral ethnic group(s), which were not mutually exclusive; owing to low cell counts, multivariable models could not include the indicators for Black, Hispanic and Middle Eastern race/ethnicity. The third sensitivity analysis stratified respondents by smoking status, grouping those who reported current or past smoking together vs. never smokers. For all continuous covariates in adjusted models, linear assumptions were evaluated and restricted spline functions were applied when non‐linear relationships were observed.

Data cleaning, analysis and figure generation were conducted in R version 4.2.2 (R Core Team 2022).

## 3 Results

Of the 27 400 women recruited to ATP between 2000 and 2015, with data on oophorectomy reported, 23 630 met inclusion criteria. The most common reason for exclusion was missing covariate data (*n* = 1345) or extreme age at menopause (*n* = 1435). Overall, 2.3% (*N* = 548) underwent a UO before experiencing natural menopause. Compared to women without a premenopausal UO, women with a premenopausal UO were more likely to have a lower parity, a history of repeated pregnancy loss or infertility, and past or current smoking status (Table 1).

**TABLE 1:** Baseline characteristics of Alberta's Tomorrow Project female participants by unilateral oophorectomy before menopause (*N* = 23 630).

Characteristic	Unilateral oophorectomy before menopause			
	No (*N* = 23 082)		Yes (*N* = 548)	
	*n*	%	*n*	%
Birth year				
1930s	728	3.2	20	3.6
1940s	4321	18.7	110	20.1
1950s	8143	35.3	245	44.7
1960s	6837	29.6	147	26.8
1970s	3039	13.2	26	4.7
Age at baseline, mean (SD)	51.3	(9.2)	52.9	(8.6)
Race/Ethnicity				
Asian ancestry	759	3.8	14	2.9
Black ancestry	120	0.6	< 10^a^	1.9
Hispanic ancestry	137	0.7	< 10^a^	0.8
Indigenous ancestry	834	4.1	24	5.0
Middle Eastern ancestry	63	0.3	0	0.0
White ancestry	19 240	95.3	459	96.2
Area of residence				
Urban	19 609	85.0	460	84.2
Rural	3453	15.0	86	15.8
Education level				
High school or less	6992	30.3	194	35.4
College degree	7007	30.4	162	29.6
University degree	6614	28.7	127	23.2
Post‐graduate degree	2468	10.7	65	11.9
Age at menarche (years), mean (SD)	12.7	(1.4)	12.5	(1.5)
Parity				
0	4071	17.6	109	19.9
1	2791	12.1	86	15.7
2	9369	40.6	190	34.7
3+	6851	29.7	163	29.7
No. of pregnancy loss				
0	15 127	65.5	309	56.4
1	5313	23.0	158	28.8
2+	2642	11.4	81	14.8
Infertility				
No	20 451	88.6	441	80.5
Yes	2631	11.4	107	19.5
Smoking status				
Never	12 522	54.3	274	50.0
Former	8426	36.5	212	38.7
Current	2134	9.2	62	11.3
Hormonal contraception use				
Never used	2290	9.9	47	8.6
Ever used	20 792	90.1	501	91.4
Duration of use (year), mean (SD)	7.9	(6.0)	7.1	(5.7)
Body mass index (BMI, kg/m^2^), mean (SD)	26.9	(5.8)	27.6	(5.9)

*Note:* 2971 missing for race/ethnicity, 22 missing for area of residence, 1 missing for education level, 322 missing for BMI, 103 missing for diabetes and 38 missing for cardiovascular disease. Proportions were calculated based on participants with available data. Race/ethnicity groups are not mutually exclusive; participants could self‐identify with more than one group.

Abbreviation: SD, standard deviation.

^a^ Cell sizes suppressed due to counts < 10.

**FIGURE 1:**
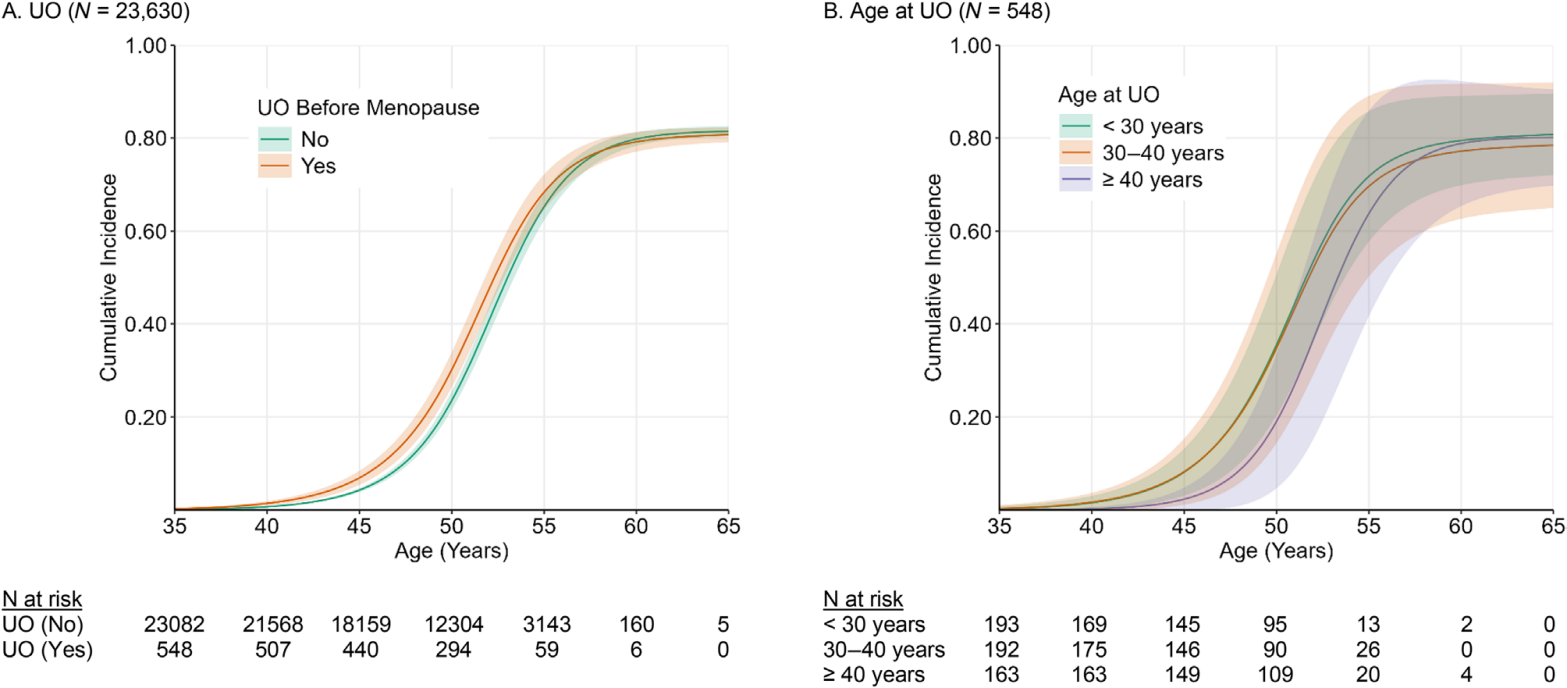
Cumulative incidence of natural menopause by UO and age at UO.

**FIGURE 2:**
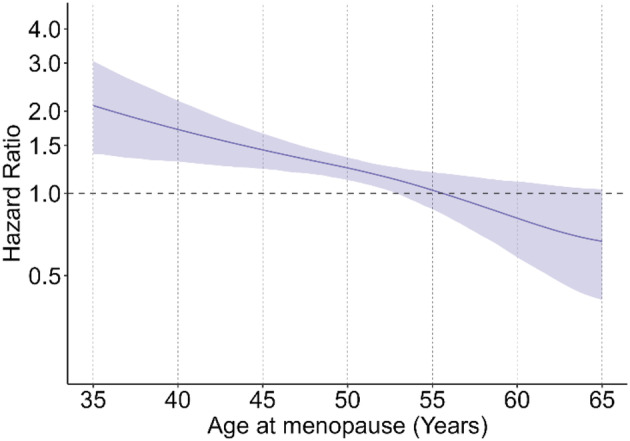
Association of UO and timing to natural menopause (*N* = 23 630). The reference group is women who did not have a UO before menopause. Adjusted models controlled for birth year, smoking, parity, age at menarche, infertility and duration of oral contraception use in years.

Overall, 57.6% of women experienced natural menopause by the end of the study. Cumulative incidence curves and flexible parametric survival models showed earlier timing of menopause among women with UO compared to those without UO (Figures 1 and 2). Adjusted HRs (AHR) indicated that this association was significant before age 53 years and time‐dependent, weakening with increasing age at menopause (age 40 AHR 1.71, 95% CI 1.31–2.19; age 50 AHR 1.24, 95% CI 1.12–1.35; age 60 AHR 0.81, 95% CI 0.58–1.10; Figure 2).

**FIGURE 3:**
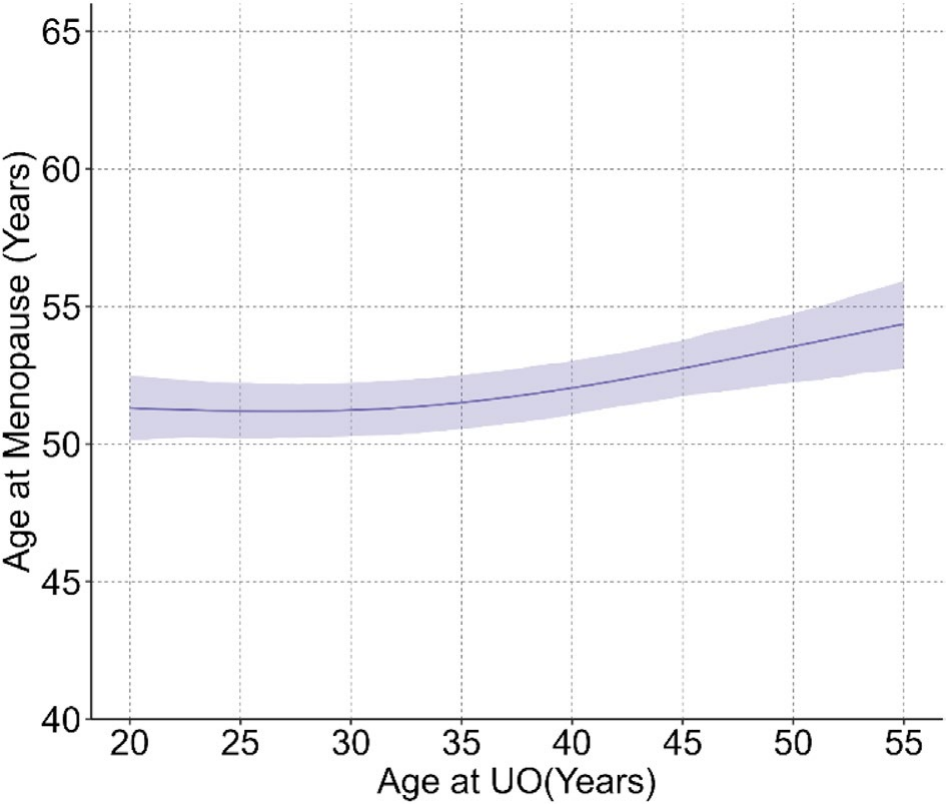
Predicted median age at natural menopause.

Among women with UO before menopause, cumulative incidence curves and flexible parametric survival models showed a non‐linear relationship between age at UO and age at menopause (Figure 1, Figure S2 and Table S2). The association was largest when UO occurred before approximately 40 years (age 20 AHR 2.27, 95% CI 1.53–3.37; age 30 AHR 2.32, 95% CI 1.46–3.54; age 40 AHR 1.86, 95% CI 1.27–2.61); and decreased in magnitude as age at UO approached the average age of natural menopause (age 45: AHR 1.53, 95% CI 1.17–1.95; age 50: AHR 1.24, 95% CI 1.08–1.40), compared to those undergoing UO at 55 years (Table S3). Predicted median age at menopause from the adjusted model was approximately 51 for those with UO between ages 20 and 40 and approximately 53 for those with UO between ages 45 and 50 (Figure 3).

Overall, 4.1% experienced early menopause (final menstrual period < 45 years old), and 0.5% experienced premature ovarian insufficiency (POI; < 40 years old). Logistic regression models showed that UO was associated with approximately four times the odds of POI (aOR: 3.75, 95% CI 1.72–8.16) and twice the odds of early menopause (aOR 1.90, 95% CI 1.30–2.79), compared to no UO (Table S1).

Results from both the survival and logistic regression models were robust to sensitivity analyses restricting to women aged ≤ 60 years at baseline, and additionally adjusting for race/ethnicity and BMI (Figures S3 and S4 and Table S4).

## 4 Discussion

### 4.1 Main Findings

This longitudinal study found that UO was associated with earlier age of menopause by 1.8 years, as well as an increased risk of early menopause and POI. This study also found that age at UO was associated with earlier onset of menopause in a non‐linear fashion. Interestingly, this association was not dependent on the age at which UO was performed, but rather characterised by a large and consistent increase in risk of earlier menopause when UO was performed between ages 20 and 40 followed by an attenuated impact of UO on risk of earlier menopause, from age 40 to 50 years.

### 4.2 Strengths and Limitations

This study is not without limitations. Foremost, we lacked data on the reasons for UO and are therefore unable to account for differences in menopause timing based upon underlying ovarian pathology resulting in UO. Given that this study focused on individuals who experienced UO without concomitant hysterectomy, the cases of UO likely represent specific ovarian pathology (such as ovarian cysts and/or torsion), rather than conditions with shared uterine pathology, such as endometriosis, or bilateral ovarian pathology, such as malignancy. Therefore, it is reasonable to assume that the contralateral remaining ovary was unlikely to be affected by pathology in the majority of these cases. The retrospective self‐reported nature of this dataset is subject to some degree of memory error, though studies have shown that the long‐term accuracy of self‐reported reproductive health data is moderate‐to‐high [22, 23, 24]. To limit recall bias, we conducted a sensitivity analysis restricting participants to those recruited < 60 years of age, demonstrating robustness of the main analysis results. We attempted to account for race/ethnicity in our analyses, which has been overlooked in prior studies possibly due to presumed heterogeneity in Japanese, Norwegian and Danish populations [16, 17, 18]. Research suggests that racial and ethnic differences exist with respect menopausal timing; [11] these may be related to various social factors, including the weathering effects of allostatic load due to socioeconomic stress, discrimination and access to healthcare [25, 26, 27, 28, 29]. Notwithstanding, ATP underrepresented women of diverse racial/ethnic groups compared to the Alberta middle‐aged female population resulting in race/ethnicity‐adjusted estimates that were imprecise and of low external validity [30].

### 4.3 Interpretation

Our findings generally align with existing studies on this topic in geographically diverse populations [16, 17, 18]. A Japanese cohort study demonstrated that females with UO experienced menopause at age 50.9 years compared to 52.1 years for females with bilaterally intact ovaries as well as documenting elevated risk of premature ovarian insufficiency (aOR 3.32 [1.42–7.77]) and early menopause before age 45 (aOR 3.94 [2.63–5.89]) [16]. These findings are echoed by a Norweigan cohort which demonstrated similar findings with females who had undergone UO experiencing younger at menopause (49.6 years compared to 50.7 years for those who had not) [18], and a Danish cohort which found a reduction in age of menopause of 1.8 years for those who had undergone UO [17]. Importantly, these prior studies used linear regression which forces a linear relationship between UO and menopause timing and inadequately captures person‐time for everyone in the sample. Our research endeavoured to address these constraints by employing flexible survival analysis, a robust analytical technique that incorporates the temporal dimension for each participant through the utilisation of censoring mechanisms. This approach ensures that the contribution of every woman in the study cohort is accounted for, irrespective of the duration of their involvement or attrition, thereby enhancing the comprehensiveness and validity of the findings. This study demonstrated a nuanced relationship between age at UO and age at final menstrual period, whereby the largest impact exists for UO before age 40, but impact is not fully diminished at any age.

The biological impact of UO on ovarian dynamics is unknown but may involve compensatory mechanisms such as ovarian growth or slowing of follicular atresia to physiologically adjust to the 50% loss of oocytes [18]. Notwithstanding, our results indicate that an earlier exhaustion of follicular reserve may still occur following UO resulting in a younger age at menopause. Our novel finding that impact of UO on timing of menopause attenuates, but does not disappear after age 40, suggests that while the contralateral ovary compensates somewhat after UO, the degree of compensation diminishes as the biologically programmed age of menopause approaches. Biological evidence supporting this finding is derived previous studies examining inter‐ovarian endocrine signalling, which have shown that women with UO have higher serum follicle stimulating hormone (FSH) and lower anti‐Müllerian hormone (AMH) levels compared to women with two ovaries [31], and required higher doses of exogenous FSH to achieve the same level of ovarian stimulation [32]. As AMH is thought to inhibit primordial follicular recruitment and subsequent atresia [33], Grynberg et al. [31] hypothesized that in women with UO, AMH produced *per follicle* would be higher compared to those with two ovaries, to limit follicular atresia in the remaining ovary, thus compensating for diminished ovarian reserve. Their findings did not provide evidence of altered per follicle AMH production, suggesting that the contralateral remaining ovary does not entirely compensate for the halved ovarian reserve pool [31]. The loss of AMH has been shown in mouse models to emancipate primordial follicles, thus resulting in more rapid pool depletion [34, 35]. It is plausible that the reason UO has less impact on the timing of menopause after the age of 40 is because AMH and Inhibin B levels have already dropped below a critical threshold resulting in more rapid follicular depletion. Therefore, UO and further AMH loss has negligible impact on the remaining follicular pool. Further studies are needed to elicit the exact mechanism by which contralateral ovarian compensation becomes exhausted.

Our model suggested that menopause occurred, on average, 2 years earlier in women with UO between ages 20 and 40. This finding represents a clinically significant shift in age at menopause, as prior work has demonstrated a 2%–3% increased risk of incident coronary heart disease, cardiovascular mortality and age‐adjusted mortality per 1 year decrease in age at menopause [36, 37, 38]. Additionally, UO is associated with poorer neurologic health, including increased risk of cognitive impairment or dementia (aOR 1.64 [1.20–22.23]) [39], as well as Parkinson disease (HR 1.68 [1.06–2.67]) [40]. While the Nurses' Health Study suggested that risk of ovarian cancer was reduced after UO (HR 0.70, [0.53–0.90]) [41], it is important to balance this against evidence that the overall effect of later menopause is an increased lifespan [42]. These studies emphasise why a shift in the age of natural menopause by even a few years is clinically significant with potential to positively impact a woman's midlife health. Additionally, our results also demonstrated that UO was associated with a substantially higher risk of early menopause < 45 years and premature ovarian insufficiency (POI) before < 40 years, though CIs were very wide given the rarity of these outcomes. POI is a significant medical condition that is associated with lower quality of life, psychological stress and high healthcare resource utilisation [43]. In addition, early menopause is often accompanied by more severe vasomotor symptoms, resulting in decreased productivity and workplace absenteeism during the peak working years [44]. Severe vasomotor symptoms are also associated with higher direct patient costs for therapies and appointments, and indirect costs to employers [44, 45, 46]. Our findings pave the way for future cost‐effectiveness studies to determine direct and indirect savings, and patient outcomes when UO is used more sparingly and subsequently the age of menopause is shifted to a more physiologic range.

It has been demonstrated that a majority of UO's are performed for non‐cancerous conditions [47]. While providers have historically offered UO to patients who have completed childbearing undergoing adnexal surgery [14], this study has important clinical and policy implications for non‐surgical or ovarian‐sparing surgical approaches to likely benign ovarian masses. Most ovarian cysts are functional and spontaneously resolve with conservative or medical management [48]. For example, when risk of malignancy is low, shared decision‐making regarding planning for ovarian cystectomy, contingent on technical feasibility, versus planned oophorectomy would include discussion of how a preserving ovarian parenchyma may benefit the patient's menopausal timing [49]. Our findings support clinical guidelines, demonstrating particular importance for patients up to age 40, independent of the patients' future fertility goals. The findings herein are in line with recent guidelines from the Society of Obstetricians and Gynecologists of Canada and the American College of Obstetricians and Gynecologists, who both recommend cystectomy with attempts at preserving ovarian parenchyma for premenopausal patients with likely benign but symptomatic ovarian masses [49, 50]. This study should also inform the booking status for ovarian torsion, as ovarian survival rate is dependent on the time between presentation and intervention [51]. Studies have shown that ovarian torsion in females has a longer decision to incision time than testicular torsion in males [51], indicating the need for increased surgeon advocacy for our female patients and elimination of gendered bias in surgical care.

## 5 Conclusion

This study demonstrated an association between UO and early menopause that is strongest when UO is performed between ages 20 and 40 and attenuated thereafter up to the average age of natural menopause. In alignment with clinical practice guidelines, this study indicates that gynaecologic providers should consider ovarian‐sparing surgeries when possible for all premenopausal women regardless of fertility goals and should urgently manage ovarian torsion to reduce the risk of necessitating performance of UO for ovarian necrosis. Future studies are needed to determine if ovarian‐sparing surgery such as cystectomy can attenuate some of these risks associated with early menopause and its downstream consequences for midlife women.

## Author Contributions

The study was conceptualised by authors E.A.B. and N.V.S., provided guidance and input towards study design, analysis and writeup. B.H. led data analysis, interpretation and figure generation and contributed to the manuscript. E.A.B. led funding approval and REB submission. R.M. contributed to manuscript writeup, data interpretation and editing.

## Conflicts of Interest

The authors declare no conflicts of interest.

## Supporting information


Data S1.

## Data Availability

Requests to access the data used in this study can be directed to the Alberta's Tomorrow Project team at ATP.Research@albertahealthservices.ca.
