# Supplementary material for: Unilateral Oophorectomy and Age at Natural Menopause: A Longitudinal Community‐Based Cohort Study
Source: BJOG. 2024 Oct 10;132(3):337–45. doi: 10.1111/1471-0528.17980 (PMC11704028; doi:10.1111/1471-0528.17980)
Supplement: Supplementary file 1 — Data S1. [file BJO-132-337-s001.docx]

**Supplementary Tables and Figures:**

Table S1: Association of UO and early menopause (< 45 years) and premature ovarian failure (< 40 years): Summary of odds ratios (N = 548)

Table S2. Association of UO and timing of natural menopause: Summary of hazard ratios

Table S3. Association of age at UO and timing of natural menopause: summary of hazard ratios (N = 548)

Table S4. Sensitivity analyses of the association of UO and early menopause (< 45 years) and premature ovarian failure (< 40 years): Summary of odds ratios

Figure S1. Flow diagram of included female participants from Alberta’s Tomorrow Project (N = 23,630)

Figure S2: Association of having UO at difference ages and timing to natural menopause (N = 548)

Figure S3. Sensitivity analyses of the association between UO / age at UO and timing to menopause

Figure S4. Predicted median age at natural menopause by smoking status

Table S1: Association of UO and EM (< 45 years) and POI (< 40 years): Summary of odds ratios

|  | No. (%) with the outcome | | Odds Ratio (95% Confidence Interval) | | | |
| --- | --- | --- | --- | --- | --- | --- |
|  |  |  | Crude | | Adjusted | |
| EM (N = 19,401) |  |  |  |  |  |  |
| No UO before 45 years (Ref) | 772 | (4.1) | 1 | – | 1 | – |
| UO before 45 years | 30 | (8.0) | 2.05 | (1.40, 3.00) | 1.90 | (1.30, 2.79) |
| POI (N = 22,190) |  |  |  |  |  |  |
| No UO before 40 years (Ref) | 108 | (0.5) | 1 | – | 1 | – |
| UO before 40 years | 7 | (2.0) | 4.09 | (1.89, 8.86) | 3.75 | (1.72, 8.16) |

EM: Early menopause, analytic sample excluded participants who ended the study or lost to follow-up before 45 years and did not experience menopause, or had menopause due to hysterectomy or removal of both ovaries before 45 years. Exposure in this analysis was defined as UO before age 45 years.

POI: Premature ovarian insufficient, analytic sample excluded participants who ended the study or lost to follow-up before 40 years and did not experience menopause, or had menopause due to hysterectomy or removal of both ovaries before 40 years. Exposure in this analysis was defined as UO before age 40 years.

Adjusted models controlled for birth year, smoking, parity, age at menarche, infertility, and duration of oral contraception use in years.

Table S2. Association of UO and timing of natural menopause: Summary of hazard ratios

| UO before menopause | Hazard Ratio (95% Confidence Interval) | | | | | |
| --- | --- | --- | --- | --- | --- | --- |
|  | Crude | | | Adjusted | | |
|  | 40 Years | 50 Years | 60 Years | 40 Years | 50 Years | 60 Years |
| No (Ref) | 1(–) | 1(–) | 1(–) | 1(–) | 1(–) | 1(–) |
| Yes | 1.83 (1.42, 2.34) | 1.26 (1.15, 1.38) | 0.77 (0.56, 1.02) | 1.71 (1.31, 2.19) | 1.24 (1.12, 1.35) | 0.81 (0.58, 1.10) |

Adjusted models controlled for birth year, smoking, parity, age at menarche, infertility, and duration of oral contraception use in years.

Table S3. Association of age at UO and timing of natural menopause: summary of hazard ratios (N = 548)

| Age at UO | Hazard Ratio (95% Confidence Interval) | | | |
| --- | --- | --- | --- | --- |
|  | Crude | | Adjusted | |
| 20 years | 2.07 | (1.41, 3.11) | 2.27 | (1.53, 3.37) |
| 30 years | 2.03 | (1.36, 3.09) | 2.32 | (1.46, 3.54) |
| 40 years | 1.66 | (1.18, 2.32) | 1.86 | (1.27, 2.61) |
| 50 years | 1.19 | (1.06, 1.34) | 1.24 | (1.08, 1.40) |
| 55 years (Ref) | 1 | – | 1 | – |

Adjusted models controlled for birth year, smoking, parity, age at menarche, infertility, and duration of oral contraception use in years.

Table S4. Sensitivity analyses of the association of UO and EM (< 45 years) and POI (< 40 years): Summary of odds ratios

| Sensitivity analysis | | No. (%) with the outcome | | Odds Ratio (95% Confidence Interval) | | | |
| --- | --- | --- | --- | --- | --- | --- | --- |
|  |  |  |  | Crude | | Adjusted | |
| 1. Restricted to women aged <= 60 years at baseline (N = 18,781) | EM (N = 15,184) |  | | | | | |
|  | No UO before 45 years (Ref) | 551 | (3.7) | 1 | – | 1 | – |
|  | UO before 45 years | 22 | (7.6) | 2.15 | (1.38, 3.35) | 1.98 | (1.27, 3.10) |
|  | POI (N = 17,626) |  | | | | | |
|  | No UO before 40 years (Ref) | 86 | (0.5) | 1 | – | 1 | – |
|  | UO before 40 years | 5 | (1.9) | 3.86 | (1.56, 9.60) | 3.58 | (1.43, 8.96) |
| 2. Additionally adjusted for race/ethnicity and BMI (N = 20,559) | EM (N = 16,876) |  | | | | | |
|  | No UO before 45 years (Ref) | 667 | (4.0) | 1 | – | 1 | – |
|  | UO before 45 years | 24 | (7.3) | 1.87 | (1.23, 2.86) | 1.74 | (1.13, 2.66) |
|  | POI (N = 19,312) |  | | | | | |
|  | No UO before 40 years (Ref) | 95 | (0.5) | 1 | – | 1 | – |
|  | UO before 40 years | 6 | (2.0) | 4.02 | (1.75, 9.25) | 3.79 | (1.64, 8.77) |
| 3(a). Stratified by smoking status – Never smokers (N = 12,796) | EM (N = 10,334) |  |  |  |  |  |  |
|  | No UO before 45 years (Ref) | 343 | (3.4) | 1 | – | 1 | – |
|  | UO before 45 years | 12 | (6.7) | 2.06 | (1.13, 3.73) | 1.98 | (1.09, 3.60) |
|  | POI (N = 12,004) |  |  |  |  |  |  |
|  | No UO before 40 years (Ref) | 38 | (0.3) | 1 | – | 1 | – |
|  | UO before 40 years | 4 | (2.4) | 7.67 | (2.70, 21.73) | 8.00 | (2.79, 22.95) |
| 3(b). Stratified by smoking status – Former and current smokers (N = 10,834) | EM (N = 9,067) |  |  |  |  |  |  |
|  | No UO before 45 years (Ref) | 429 | (4.8) | 1 | – | 1 | – |
|  | UO before 45 years | 18 | (9.1) | 1.98 | (1.21, 3.24) | 1.88 | (1.14, 3.08) |
|  | POI (N = 10,186) |  |  |  |  |  |  |
|  | No UO before 40 years (Ref) | 70 | (0.7) | 1 | – | 1 | – |
|  | UO before 40 years | 3 | (1.6) | 2.34 | (0.73, 7.50) | 2.32 | (0.72, 7.49) |

EM: Early menopause; POI: Premature ovarian insufficiency.

Adjusted models controlled for birth year, smoking, parity, age at menarche, infertility, and duration of oral contraception use in years.

Sensitivity analysis 2 additionally controlled for race/ethnicity (Asian, Black, Indigenous, Hispanic, Middle Eastern, and White) and BMI. Black, Hispanic and Middle Eastern were not included in one of or both models due to small cells.

Figure S1. Flow diagram of included female participants from Alberta’s Tomorrow Project (N = 23,630)

| 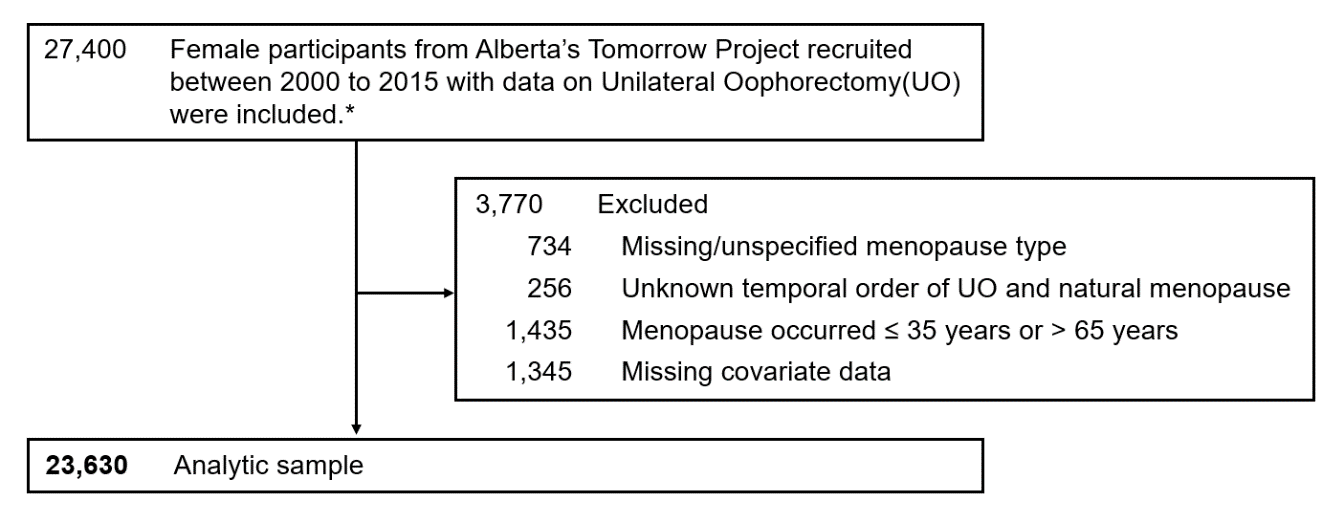 |
| --- |

*Participants whose last survey was one of CORE, UHLQ, and S17 provided data on whether they had a UO and the age when they had the procedure.

Figure S2: Association of having UO at difference ages and timing to natural menopause (N = 548)

| 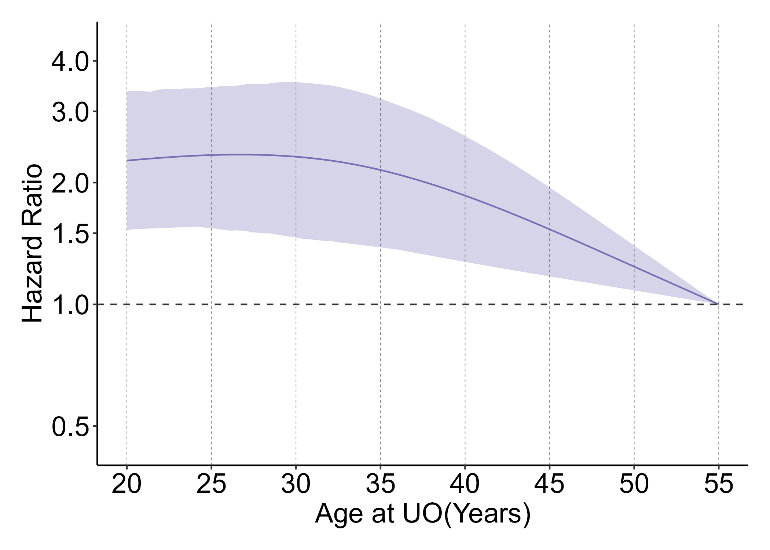 |
| --- |

The reference is having a UO at 55 years (before menopause).

Adjusted models controlled for birth year, smoking, parity, age at menarche, infertility, and duration of oral contraception use in years.

Figure S3. Sensitivity analyses of the association between UO / age at UO and timing to menopause

| Sensitivity analysis | (A) UO and timing to menopause | (B) Age at UO and timing to menopause |
| --- | --- | --- |
| 1. Restricted to women aged <= 60 years at baseline (N = 18,781) | 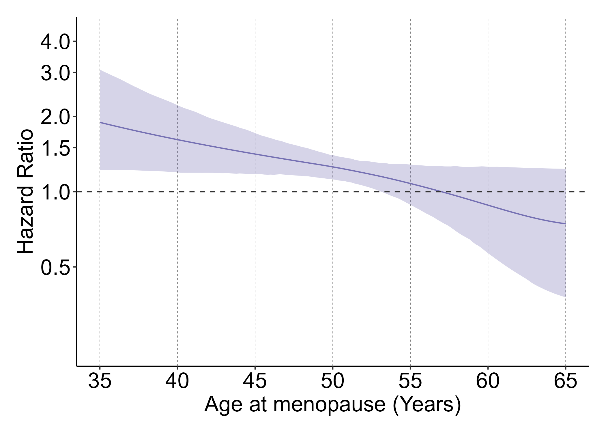 | 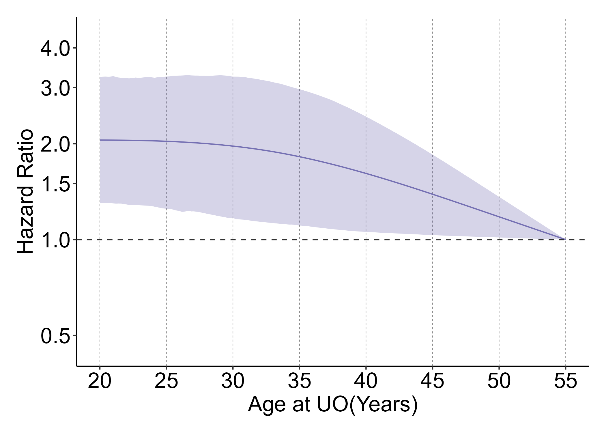 |
| 2. Additionally adjusted for race/ethnicity and BMI (N = 20,559) | 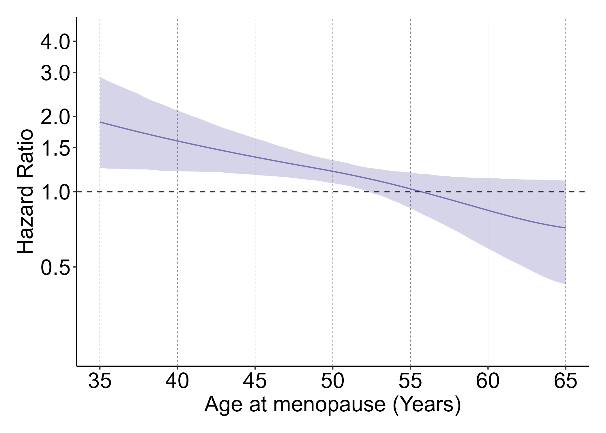 | 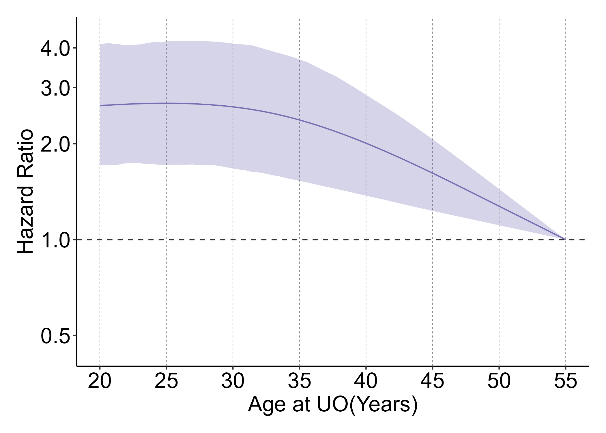 |
| 3(a). Stratified by smoking status – Never smokers (N = 12,796) | 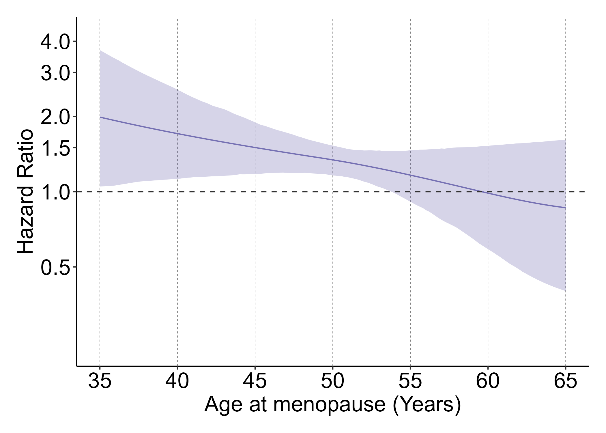 | 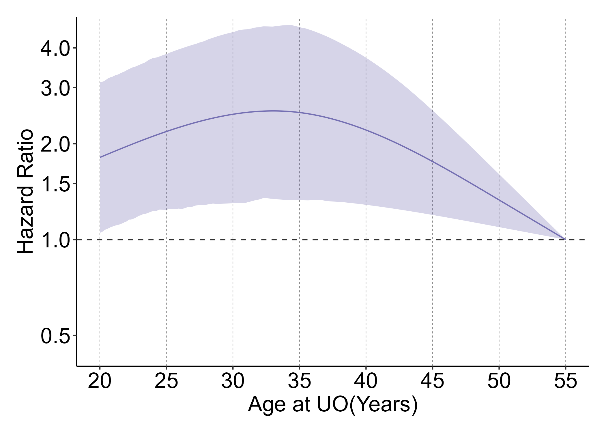 |
| 3(b). Stratified by smoking status – Former and current smokers (N = 10,834) | 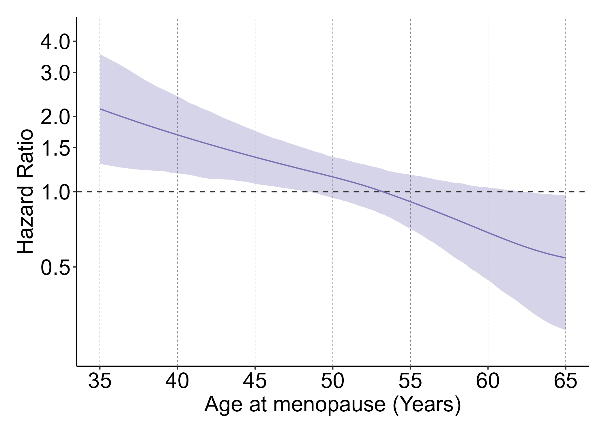 | 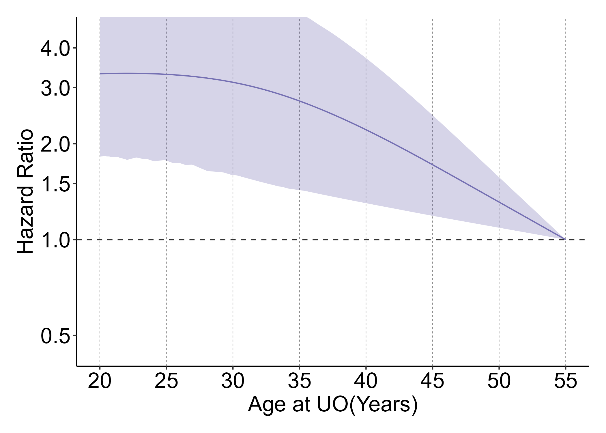 |

Adjusted models controlled for birth year, smoking, parity, age at menarche, infertility, and duration of oral contraception use in years.

Sensitivity analysis 2 additionally controlled for race/ethnicity (Asian, Black, Indigenous, Hispanic, Middle Eastern, and White) and BMI. Black, Hispanic and Middle Eastern were not included in one of or both models due to small cells.

Figure S4. Predicted median age at natural menopause by smoking status

| A. Non-smokers (N = 12,796) | B. Former smokers (N = 8,638) | C. Current smokers (N = 2,196) |
| --- | --- | --- |
| 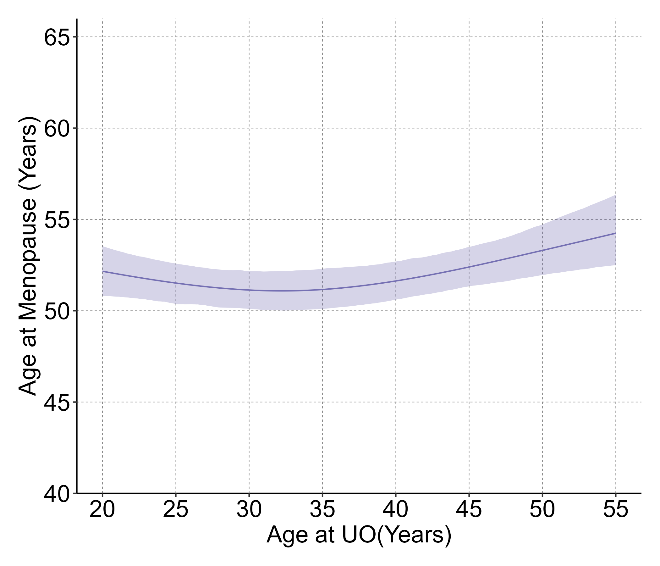 | 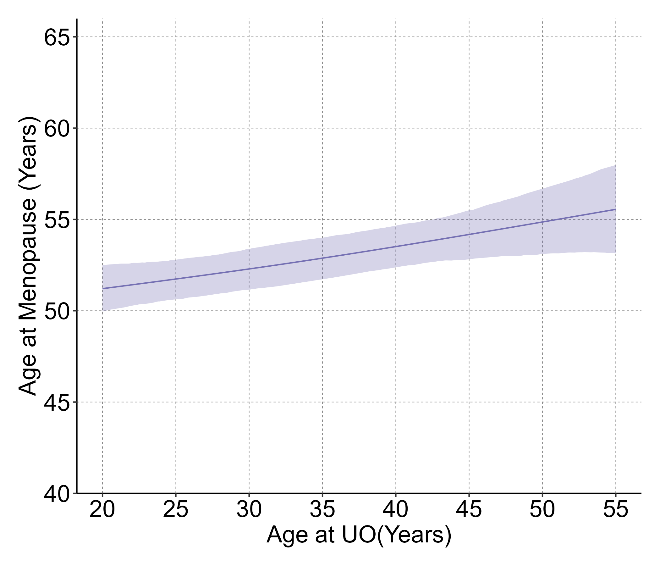 | 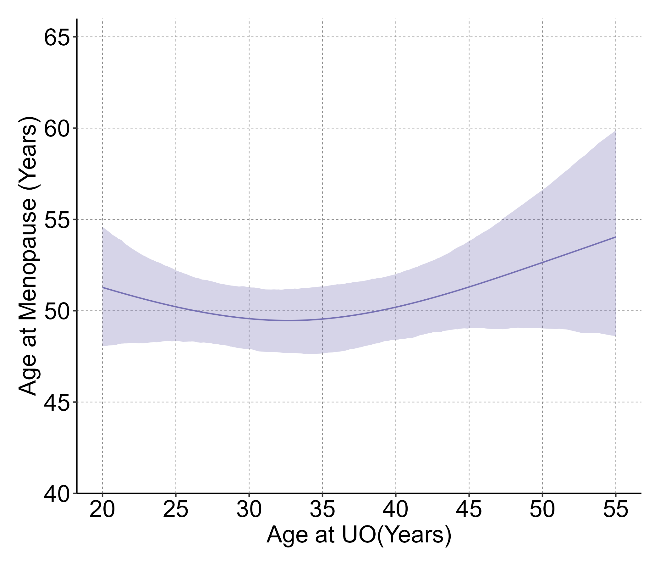 |
